# Jwalk and MNXL web server: model validation using restraints from crosslinking mass spectrometry

**DOI:** 10.1093/bioinformatics/bty366

**Published:** 2018-05-07

**Authors:** Joshua M A Bullock, Konstantinos Thalassinos, Maya Topf

**Affiliations:** 1 Institute for Structural and Molecular Biology, Birkbeck College, University of London, London, UK; 2 Institute for Structural and Molecular Biology, UCL, University of London, London, UK

## Abstract

**Motivation:**

Crosslinking Mass Spectrometry generates restraints that can be used to model proteins and protein complexes. Previously, we have developed two methods, to help users achieve better modelling performance from their crosslinking restraints: Jwalk, to estimate solvent accessible distances between crosslinked residues and MNXL, to assess the quality of the models based on these distances.

**Results:**

Here, we present the Jwalk and MNXL webservers, which streamline the process of validating monomeric protein models using restraints from crosslinks. We demonstrate this by using the MNXL server to filter models made of varying quality, selecting the most native-like.

**Availability and implementation:**

The webserver and source code are freely available from jwalk.ismb.lon.ac.uk and mnxl.ismb.lon.ac.uk.

**Supplementary information:**

Supplementary data are available at *Bioinformatics* online.

## 1 Introduction

Crosslinking Mass Spectrometry (XL-MS) is an experimental method that can generate sparse structural information on proteins, complementary to traditional structural techniques. Briefly, an XL-MS experiment consists of (a) crosslinking of your target protein (or protein complex); (b) digesting the crosslinked protein and (c) identifying via MS which residues are crosslinked. This results in restraining information that can then be used to model the protein of interest.

Jwalk (Bullock *et al.*, 2016) is a program that calculates the Solvent Accessible Surface Distance (SASD), which is defined as the shortest distance between two residues across the surface of the protein (Kahraman *et al.*, 2011). The SASD is a theoretically more correct approximation of the distance between crosslinked residues than the commonly used Euclidean distance, because the Euclidean distance permits travelling through the protein mass whereas a crosslinker cannot travel through protein mass. Other methods exist that calculate the SASD, while also considering side-chain flexibility (Degiacomi *et al.*, 2017).

Crosslinks can also act as a proxy for solvent accessibility, as residues must be solvent exposed if they are to be crosslinked. Utilizing this extra information, we created the crosslink scoring function called *Matched and Non-accessible Crosslink Score* (MNXL), which we found to outperform more conventional methods when scoring protein monomers (Bullock *et al.*, 2016). In order to streamline the XL-MS modelling procedure, we have incorporated these two developments (MNXL and Jwalk) into two webservers. Both programs are also standalone and freely available to download.

## 2 Implementation

**Fig. 1.**
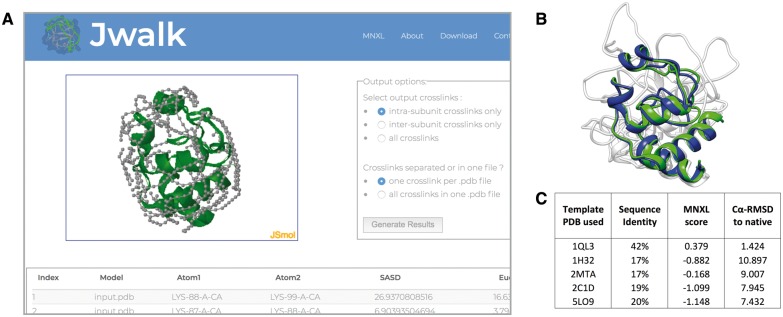
(**A**) Partial screenshot of the Jwalk result page with PDB id. 1HRC shown. (**B**) The models of the test-case superposed in grey, with the best scoring model based on PDB id. 1QL3 (blue) and the native 1HRC (green). (**C**) Results table showing MNXL is able to select the lowest Cα-RMSD model

Jwalk and MNXL are written in Python 2.7. For a full description of Jwalk see (Bullock *et al.*, 2016). Jwalk outputs a list of all the SASDs and Euclidean distances between target residues in a *.txt* file along with a .*pdb* file that contains all the SASD paths modelled using glycine pseudo atoms. On the Jwalk webserver (Fig. 1A), SASD paths are visualized with JSmol (Hanson *et al.*, 2013). MNXL takes as input a list of experimental crosslinks and Jwalk *.txt* output files (which can also be provided by the user independently). The SASDs of the experimental crosslinks are then scored using the MNXL scoring function (Bullock *et al.*, 2016). Using the webserver, users can also go directly from *.pdb* file to MNXL score (instead of running Jwalk separately).

MNXL outputs the scores for each model in a *.txt* file. Higher scores indicate better models. Additionally, MNXL outputs the number of crosslinks that are matched, violating and non-accessible to aid model assessment. The source code for both Jwalk and MNXL is available under a Creative Commons license at http://topf-group.ismb.lon.ac.uk/Software.html.

## 3 Results

To demonstrate the utility of the Jwalk/MNXL web server, we show the ability for the combination of Jwalk and MNXL to filter comparative models made with different templates. Five different models of the horse heart cytochrome C crystal structure (PDB id: 1HRC) were made with MODELLER (Eswar *et al.*, 2007) using templates of various quality, taken from the HHPred server (Alva *et al.*, 2016) with probability score > 96% in all cases (Fig. 1B). The five template PDB ids (and associated sequence identity) are: 1QL3 (42%), 5LO9 (20%), 1H32 (17%), 2MTA (17%) and 2C1D (19%), respectively. These comparative models were then uploaded into the MNXL webserver [along with the experimentally observed crosslinks taken from XLdb (Kahraman *et al.*, 2013)]. The MNXL score was able to successfully select the model made using template 1QL3, which is the model with the lowest Cα-RMSD to the native structure (Fig. 1C)––for further discussion of the results see Supplementary Material.

## 4 Conclusion

We have created webservers for MNXL and Jwalk, two methods that can be used to validate models using restraints from XL-MS and demonstrated how it can be useful in filtering comparative models built from different templates. These webservers are designed to be user-friendly, in order to make it easier for the novice user to make better use of their crosslinking data. We hope to expand these platforms to incorporate the modelling of protein complexes in the near future.

## Supplementary Material

Supplementary Data
