# Supplementary material for: Jwalk and MNXL web server: model validation using restraints from crosslinking mass spectrometry
Source: Bioinformatics. 2018 May 7;34(20):3584–5. doi: 10.1093/bioinformatics/bty366 (PMC6184817; doi:10.1093/bioinformatics/bty366)
Supplement: Supplementary Data [file bty366_supplemental_data.docx]

Supplementary Data

Results

To generate comparative models for horse heart cytochrome c (PDB id: 1HRC) its sequence was first uploaded onto HHPred (ref), where 5 templates were chosen at a range of different sequence identities (42 - 19%) (PDB ids: 1QL3, 5LO9, 1H32, 2MTA, and 2C1D). Models were then generated using the MODELLER (ref) pipeline on the HHPred server. A single model for each template was generated. In three cases, the models were renumbered so that all models had the same sequence and residue numbers. This is crucial when modelling with the MNXL server as the server does not check that sequence numbering is equivalent. The models were simultaneously uploaded onto the server and scored using a dataset of 48 cross-links (the cross-link data file used on the server can be found below). MNXL was able to select the model with the lowest Cα-RMSD to the X-ray structure 1HRC, which was based on the template PDB id: 1QL3 (Figure A, Figure S1)

The MNXL score details the number of matched, violating and non-accessible cross-links for each model, which can give insights into how the cross-linking information filters the models. We previously found non-accessible cross-links to play an important role in modelling protein monomers; this is again confirmed here because the best model can be chosen on a basis of non-accessible cross-links alone (Figure S1).

Scoring the X-ray structure 1HRC with MNXL reveals that 10 cross-links have an SASD over 33 Å, suggesting significant protein flexibility. This is likely why there is not a direct correlation between Cα-RMSD and MNXL across the models. The model made using the template 1QL3 has only 6 violating cross-links, however, 1HRC is still scored the highest by MNXL. This shows the importance of using all three scoring terms, rather than just the Number of Violations, when filtering different structural models using cross-linking information.


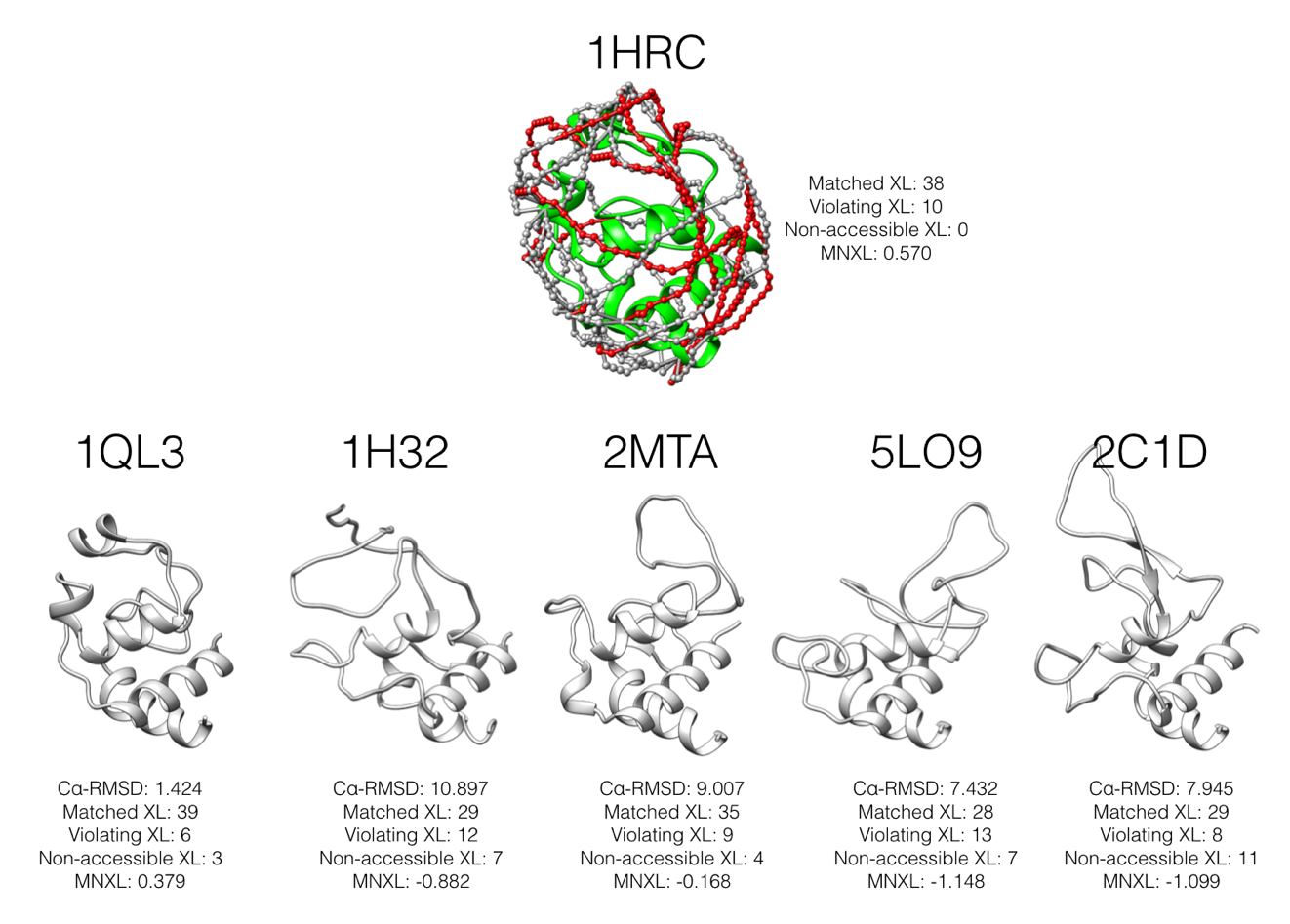


Figure S1 | The native structure 1HRC in green with the Matched SASDs shown in grey and the Violating SASDs shown in red. Below are the 5 models scored on the MNXL web-server (each model generated using a template indicated by the PDB id). Each model has its Cα-RMSD from the X-ray structure (PDB id: 1HRC), as well as the number of Matched, Violating and Non-accessible cross-links, and the MNXL score.

Crosslinking reference file

Below is the cross-link reference file used for scoring the 5 models:

1|A|38|A|

1|A|52|A|

1|A|4|A|

1|A|87|A|

38|A|52|A|

38|A|87|A|

4|A|38|A|

86|A|87|A|

24|A|26|A|

4|A|86|A|

98|A|99|A|

21|A|24|A|

38|A|99|A|

52|A|54|A|

54|A|59|A|

4|A|7|A|

59|A|78|A|

71|A|98|A|

71|A|72|A|

71|A|78|A|

72|A|78|A|

72|A|87|A|

6|A|26|A|

6|A|7|A|

85|A|86|A|

85|A|87|A|

86|A|99|A|

7|A|99|A|

7|A|12|A|

7|A|86|A|

38|A|54|A|

38|A|59|A|

52|A|78|A|

54|A|71|A|

54|A|72|A|

59|A|85|A|

71|A|85|A|

72|A|85|A|

87|A|98|A|

21|A|99|A|

26|A|72|A|

26|A|78|A|

26|A|85|A|

38|A|71|A|

52|A|72|A|

4|A|12|A|

6|A|38|A|

86|A|98|A|
